# Role of poultry meat in a balanced diet aimed at maintaining health and wellbeing: an Italian consensus document

**DOI:** 10.3402/fnr.v59.27606

**Published:** 2015-06-09

**Authors:** Franca Marangoni, Giovanni Corsello, Claudio Cricelli, Nicola Ferrara, Andrea Ghiselli, Lucio Lucchin, Andrea Poli

**Affiliations:** 1 Nutrition Foundation of Italy (NFI), Milan, Italy; 2 Department of Health Promotion and Mother and Child, University of Palermo, Palermo, Italy; 3 Italian Society of General Medicine (SIMG), Florence, Italy; 4 Department of Translational Medical Sciences, University of Naples ‘Federico II’, Naples, Italy; 5 Scientific Institute of Telese (BN), Salvatore Maugeri Foundation, IRCCS, Naples, Italy; 6 Agriculture Research Council-Food and Nutrition Research Centre (CRA-NUT), Rome, Italy; 7 Italian Association of Dietetics (ADI), Dietetics and Clinical Nutrition Unit, Bolzano Health District, Bolzano, Italy

**Keywords:** human nutrition, poultry meat consumption, poultry meat composition, balanced diet

## Abstract

The relationship between the consumption of meat and health is multifaceted, and it needs to be analyzed in detail, with specific attention to the relevant differences that characterize the effects of the different meat types, as yet considered by only a limited literature. A variable but moderate energy content, highly digestible proteins (with low levels of collagen) of good nutritional quality, unsaturated lipids (mainly found in the skin and easily removed), B-group vitamins (mainly thiamin, vitamin B6, and pantothenic acid), and minerals (like iron, zinc, and copper) make poultry meat a valuable food. Epidemiological studies performed across the world, in highly diverse populations with different food preferences and nutritional habits, provide solid information on the association between poultry consumption, within a balanced diet, and good health. Consumption of poultry meat, as part of a vegetable-rich diet, is associated with a risk reduction of developing overweight and obesity, cardiovascular diseases, and type 2 diabetes mellitus. Also, white meat (and poultry in particular) is considered moderately protective or neutral on cancer risk. The relevance of poultry meat for humans also has been recognized by the UN Food and Agricultural Organization (FAO), who considers this widely available, relatively inexpensive food to be particularly useful in developing countries, where it can help to meet shortfalls in essential nutrients. Moreover, poultry meat consumption also contributes to the overall quality of the diet in specific ages and conditions (prior to conception, during pregnancy up to the end of breastfeeding, during growth, and in the geriatric age) and is suitable for those who have an increased need for calorie and protein compared to the general population.

When gauging the relationship between nutrition and health, animal-derived foods (and meat in particular) are usually assessed in a global manner: the lay public perception of their actions is consequently often quite negative. However, various aspects of this relationship deserve to be analyzed in detail, in particular the relevant differences that characterize the health effects of different meat types. Only a few studies, in fact, have considered the impact of these foods on health in relation to the animal species of origin (1–4); in many cases, the distinction is simply made between red and white meat (5–7). Nonetheless, the literature focused on poultry meat has identified many positive aspects, from a nutritional point of view (8–10), associated with its regular consumption.

The aim of this consensus document is to review the available evidence on the association between poultry meat intake, diet quality, and general health status. In fact, a number of observational studies and meta-analyses have been published on these topics; these data represent the source of information on which this consensus document has been based.

## Macro- and micronutrient composition and energetic value of chicken and turkey meat

Meat and its derived products provide relevant quantities of essential nutrients at higher concentrations compared with other foods. The nutrient content in the animal's musculature does not vary significantly between species, whilst the ratio between fat and muscle mass in the edible part does vary considerably. The quality of animal fat and the amounts of nutrients largely depend on the animal's diet or its genetic pattern, despite the fact that recent specific farming techniques (organic, free range) have been shown to influence some compositional aspects of meat (specifically, poultry meat). Cooking and heating processes usually have only minimal effects on the nutritional profile of meat, mostly corresponding to the concentration of nutrients (including fat) and a decrease in water content.

In particular, the energetic value of poultry meats varies between chicken breast and chicken thighs with skin (Table 1) (11): the presence of skin (due to its fat content) increases the caloric value by around 25–30%. It must be noted that cooking also affects energetic value, which increases by 30–50% for meat with skin (essentially due to a loss of water during the cooking process) (12).

**Table 1:** Nutrient composition of some raw and cooked (roasted) cuts of chicken meat^a^

			Fats (g)				
			
	En kcal	Proteins (g)	Total	Saturated	Monounsaturated	Polyunsaturated	Cholesterol (mg)
Whole chicken with skin, raw	171	19.0	10.6	3.27	4.12	2.29	93
Whole chicken with skin, roasted^b^	200	27.1	10.2	3.04	2.91	2.66	119
Whole chicken with skin, roasted [rotisserie]	246	28.3	14.7	4.38	4.19	3.83	119
Whole chicken skinless, raw	110	19.4	3.6	1.23	1.08	0.81	75
Whole chicken skinless, roasted^b^	160	27.9	5.4	1.72	1.38	1.51	109
Whole chicken skinless, roasted [rotisserie]	206	28.9	10.0	3.19	2.56	3.80	109
Chicken, wing skinless, raw	193	20.3	12.4	4.24	3.72	2.79	89
Chicken, wing with skin, raw	196	16.7	14.3	4.41	5.56	3.09	82
Chicken, wing with skin, roasted^b^	283	31.7	17.4	5.46	6.45	3.84	91
Chicken, drumstick with skin, raw	125	18.4	5.7	1.61	1.61	1.58	94
Chicken, drumstick with skin, roasted^b^	201	31.2	8.5	2.53	2.43	2.22	91
Chicken, drumstick skinless, raw	107	18.5	3.7	1.08	1.06	0.98	88
Chicken, drumstick skinless, roasted^b^	175	29.9	6.2	1.98	1.58	1.73	109
Chicken breast, raw	100	23.3	0.8	0.25	0.19	0.23	60
Chicken breast, roasted^c^	129	30.2	0.9	0.29	0.23	0.25	75
Whole turkey with skin, raw	135	18.2	6.9	2.22	1.66	2.96	195
Whole turkey skinless, raw	109	21.9	2.4	0.90	0.62	0.60	63
Turkey rump, raw	107	24.0	1.2	0.38	0.31	0.34	50
Turkey rump, roasted^c^	131	29.6	1.4	0.43	0.37	0.38	62
Turkey drumstick with skin, raw	126	17.9	6.0	1.91	1.81	1.70	73
Turkey drumstick with skin, roasted^b^	191	26.7	9.3	2.80	2.67	2.71	110
Turkey drumstick skinless, raw	113	18.0	4.6	1.72	1.18	1.15	67
Turkey drumstick skinless, roasted^b^	190	28.0	8.7	2.84	2.41	2.48	107

a From Missmer et al. (1).

b Oven-roasted, fat-free; ^c^sautéed without seasoning.

### Protein

Poultry meat, like other meats, milk, and eggs, has a protein component usually defined as ‘high quality’. Animal-derived foods have a Protein Digestibility Corrected Amino Acid Score (PDCAAS) value equivalent to or slightly below one (13). Conversely, plant-derived foods, which – despite containing a relevant quantity of protein – have a less favorable protein profile (they are generally lacking in one or more essential amino acids and/or more difficult to digest), have a substantially lower PDCAAS value (e.g. 0.75 for beans and 0.5 for wheat).

Of all macronutrients, proteins are the minor contributors to the daily caloric intake. It is also noteworthy that protein is the only macronutrient for which, similar to micronutrients, a precise recommended intake has been established.

According to the European Food Safety Authority (EFSA) Daily Reference Values (14), as an example, the average recommended daily intake of protein (i.e. the minimum consumption level required to satisfy the recommended intake for 50% of healthy subjects for adults (both men and women)) is 0.66 g protein/kg body weight per day based on nitrogen balance data, and reaches up to 1.12 g per kg of body weight for infants.

The reference dietary intake for the population (the population reference intake, or PRI, which is equivalent to the sufficient serving size required to insure the coverage of almost all (97.5% of) healthy subjects) is, of course, set at higher levels: it was estimated to be 0.83 g protein/kg body weight per day, applicable both to high-quality protein and to protein in mixed diets, for adults of all ages. Such levels are subject to a progressive increase throughout the three trimesters of pregnancy, according to weight gain, as well as during breastfeeding. Similar values are reported as Dietary Reference Intakes for the American population.

It is commonly assumed that the recommended protein intake also increases for men and women over 65 years of age in order to counteract sarcopenia, which occurs frequently in the elderly. Based on the analysis of the collected data in the context of the National Health and Nutrition Examination Survey (NHANES) III study on more than 6,300 men and women, in this stage of life, protein intake should equal about 1.2–1.3 g/kg/day, and it should be especially from protein of high biological value (15).

According to the EFSA, the available information on protein health effects is sufficient to establish the minimal levels, which essentially correspond to the amount of nitrogen required to maintain an equal balance, but not to set the maximal tolerable levels of protein. The EFSA suggests that an intake equivalent to twice the PRI can be considered safe. For example, this would correspond to 92.4 g of protein for an adult weighing 70 kg and following a moderately active lifestyle, equal to about 15% of the total calories of a 2,500 kcal diet.

The protein content of most meat (including poultry meat) ranges between 15 and 35%, depending on the water and fat content of the product. Cooking also causes an increase in protein concentration, which reaches up to 60% in weight for skinless turkey drumstick and skinless chicken drumstick.

The low content of collagen (a structural protein) is another favorable characteristic of poultry meat. Collagen reduces the digestibility of meat, and high levels of this protein in muscular meat are associated with a lower percentage of digested product per unit of time.

### Fats

Meat contributes to fats, especially saturated ones; its consumption is therefore potentially associated with an excess intake of these nutrients and the corresponding negative health consequences. Nonetheless, the suggested dietary target for fats in the general healthy population ranges from 25 to 35% of total energy, so that a typical average intake of 2,000 kcal results in 70 or more grams of these nutrients per day. In addition, when consumed in appropriate quantities (i.e. compatible with a healthy balanced diet), fat plays a number of important roles: it provides ‘essential fatty acids’ (such as linoleic and alpha-linolenic acids) and lipophilic vitamins (A, D, E, and K); it represents a major source of energy; it promotes a sense of satiety due to slowing effects on gastric emptying; it reduces, for the same reason, the bioavailability of carbohydrates (and, hence, the glycemic response); and, finally, it enhances the taste, smell, and texture of foods.

It must also be noted that the muscular part of animals, lacking visible fat, has a fairly limited lipid content, which was further reduced over the past decades, thanks to the progress in farming techniques and feed quality and profile.

Lipid intake associated with poultry meat is variable and dependent on the cut considered. Fats are nonetheless mainly found in skin and can, therefore, be easily removed (Table 1). The lipid content of chicken and turkey is around 1% in the leanest cuts, such as chicken breast and turkey rump, and around 17% (at the opposite extreme) in cooked chicken wings with skin. The inclusion of skin can increase these values.

Cooking can also increase the fat content concentration (although less so compared to protein content), by removing water from meat, or by adding fats present in the condiments used during preparation (as for ‘rotisserie’ roast chicken). Nonetheless, when compared to other types of meat, poultry appears to be relatively low in fat.

From a nutritional point of view, the composition of poultry fat is favorable: it includes significant amounts of monounsaturated fatty acids (only a third of total fat is made up of saturated fatty acids) (Table 1) and, in comparison with bovine, ovine, or pig meat, substantial amounts of polyunsaturated fats, especially the omega-6 or n-6 linoleic acid (18:2 n-6) and arachidonic acid (20:4 n-6), which can be found mostly in the skin (Table 2) (16). Thanks to vegetable-derived feed, rich in alpha-linolenic acid (a precursor of long-chain omega-3 or n-3 fatty acids), poultry also provides some amount of this class of fats. In most Western countries, where fish consumption (a major source of omega-3) is relatively low, poultry meat may thus represent an important source of these fatty acids (17).

**Table 2:** Content of *n*-6 and *n*-3 polyunsaturated fatty acids (mg/100 g) in selected meats

	*n*-6		*n*-3			
	
	LA 18:2*n*-6	AA 20:4*n*-6	ALA 18:3*n*-3	EPA 20:5*n*-3	DPA 22:5*n*-3	DHA 22:6*n*-3
Poultry meat	1,443	98	73	5	18	25
Chicken, with skin	2,880	80	140	10	10	30
Chicken, without skin	550	80	20	10	20	30
Turkey with skin	1,700	110	110	0	20	20
Turkey, without skin	640	120	20	0	20	20
Pork	831	68	53	3	7	2
Egg	1,272	156	31	0	6	44
Bovine meat	277	24	105	5	8	4
Beef rib eye	240	20	10	NA	NA	NA
Beef sirloin	94	9	20	5	15	10
Goat and mutton	460	64	178	5	19	21
Lamb	369	84	54	5	7	10

Modified from Sinha et al. (5).

### Carbohydrates

Animal-derived foods contain very few carbohydrates, which, conversely, are found abundantly in plant-based foods. The only naturally occurring carbohydrate in muscle is glycogen, whose content rapidly decreases following butchering. In certain cured meats, sucrose or glucose are added during the manufacturing process.

### Vitamins and minerals

Meat represents an excellent source of the majority of hydrophilic vitamins, and it is the ideal dietary source of vitamin B12. The amounts of B-group vitamins (e.g. niacin, vitamin B6, and pantothenic acid) in poultry are very similar to those of other meats and do not significantly diminish during cooking. While red meat is the most abundant in terms of vitamin B12, poultry supplies an important amount of niacin. Lipophilic vitamins such as vitamins E and K, contained in muscles, are less abundant in meat compared to plant-based foods.

Meat also provides several minerals. As shown in Table 3, despite a large variability in iron concentration across different types of meat, poultry also provides this mineral (100 g of chicken thighs provide 1.4 mg of iron, compared to 1.3 mg for an equal amount of rump steak from an adult bovine) (18).

**Table 3:** Total iron, heme iron, and non-heme iron content in raw and cooked poultry meats (mg/100 g) (fresh weight)

	Raw			Cooked		
	
	Total Fe	Heme Fe	Non-heme Fe	Total Fe	Heme Fe	Non-heme Fe
Chicken (mean)	0.59±0.1	0.22±0.1	0.37±0.2	1.01±0.3	0.28±0.1	0.73±0.3
Turkey (mean)	0.79±0.2	0.35±0.1	0.44±0.1	1.25±0.4	0.45±0.2	0.80±0.2
Beef (mean)	2.09±0.2	1.82±0.2	0.28±0.4	3.39±0.4	2.63±0.5	0.77±0.2
Veal fillet	0.85±0.3	0.71±0.3	0.14±0.6	1.58±0.4	1.33±0.6	0.25±1.0
Rabbit	0.45±0.1	0.25±0.1	0.20±0.2	0.60±0.1	0.31±0.1	0.29±0.2
Pork (mean)	0.42±0.1	0.26±0.1	0.17±0.2	0.64±0.2	0.39±0.2	0.24±0.1

Modified by Oostindjer et al. (7).

Sodium is only minimally present in fresh meat and in poultry too, and does not significantly contribute to total dietary intake. Processed meat products, on the other hand, can contain high or very high quantities of sodium, added as a preservative or flavor enhancer.

Chicken meat is also an excellent source of selenium. Moreover, lean meat contains factors that promote the bioavailability of a variety of nutrients, which is hence often larger compared to that of the same nutrients present in plant-based foods. Besides heme iron, zinc, copper, and B vitamins are also highly bioavailable when consumed with meat. At the same time, meat also promotes the bioavailability of nutrients found in other foods when consumed concurrently. For example, the absorption of non-heme iron contained in other foods is increased when they are consumed with meat.

## Poultry meat: levels of consumption

According to FAO data, the consumption of poultry meat, like all other types of meat, has progressively increased from the past century to today in Europe and in the USA and has generally remained stable over the past years.

European behavior with regard to dietary consumption in general and poultry consumption, in particular, is considerably different than that of the United States. The NHANES results (19) confirm that in the USA, the shift in consumption from red meat to white meat was higher than in any other country. Nonetheless, red meat still represents the majority of meat consumed in the USA (58%), while processed meats occupy about 22% of the market. According to the study, in 2003–2004, the total intake of meat in the American diet was equivalent to an average of 128 g per day, with a large variability in type and quantity of meat, also based on education, age, and gender. This survey has shown that meat consumption in the United States is therefore around three times higher compared to the global mean: this aspect should be taken into proper consideration when defining the sanitary policies oriented toward the reduction of the prevalence of chronic diseases.

In Europe, data on poultry meat consumption originating from the European Prospective Investigation into Cancer and Nutrition (EPIC) study (20) provide values which differ across several geographical areas: total meat consumption reaches a maximum in Spain (126.9 g per day in San Sebastian) and a minimum in Greece (45.6 g per day), while daily intakes of poultry meat vary from 7.6 g in Umea (Sweden) to 29.2 g in San Sebastian. In Italian population groups, the average daily intake of poultry meat is of about 20 g (chicken representing 65% of total poultry meat), with a peak of 23.4 g for subjects recruited in the Center (Florence), and the lowest levels (14.6 g) for residents in the South (Naples).

Similar consumption levels of poultry meat have been registered in the representative sample of the Italian population recruited for the INRAN-SCAI 2005–2006 survey (21).

As reported in the FAO database, poultry meat represents less than 30% of the meat in the Italian diet, which is more abundant in cured meats, sausages, and other processed products.

## Poultry consumption and human health

Epidemiological studies conducted across various parts of the world, in highly diverse populations, with different food preferences and nutritional habits, provide solid information on the association between diet and health. Several prospective studies support the association between poultry consumption, within a balanced diet, and a reduction in the risk of developing cardiovascular (CV) diseases and their risk factors, such as overweight and insulin resistance, and tumors.

### Weight control/obesity

The benefits of protein consumption, including animal proteins, in weight management are supported by observational studies and have been the object of intervention trials, which yielded mixed results (22).

An analysis of 15 randomized controlled studies, with a follow-up ranging from 1 week to 1 year (23), comparing the effects of low- to high-protein diets on body weight, showed a statistically significant difference in weight loss between the two groups in the majority of cases, in favor to the higher protein intake. Only very few small-scale studies provide contradicting results, probably due to the different compliance of the enrolled subjects (24). There is evidence that in the short term (i.e. up to 6 months), weight loss increased in hypocaloric, high-protein diets if compared with hypocaloric diets with low protein content (25). The possible mechanisms responsible for this effect include increased satiety, followed by a lower calorie intake during subsequent meals and decreased carbohydrate consumption, within dietary regimens containing a higher proportion of protein (24, 26). It was also hypothesized that these mechanisms could in some way be synergistic. In addition to their satiety producing effect, yielding a subsequent reduction in energy intake, proteins are also responsible for higher thermogenesis (by increasing protein synthesis and adenosine triphosphate expense linked to peptide bond formation, as well as urea production and gluconeogenesis) (27).

The intake of one serving of protein in substitution of the same amount of carbohydrates decreases the overall glycemic load of the meal (28).

On the other hand, very high intakes of meat have been associated with increases in body weight. In the previously mentioned EPIC study, as an example, an increase of 250 g in daily meat consumption (including all meat types) was associated with an extra 2 kg weight gain over 5 years, in both normal-weight and overweight men and women (22). It is worth mentioning that 250 g/day (1.750 kg of meat per week) is a particularly large serving size, corresponding to about 450 additional kcal per day; this is currently considered as incompatible with any weight control strategy and is very infrequent in European countries (29).

### Cardiovascular diseases

Also, as concerns CV health, the effect of protein intake seems to be dependent upon dietary sources (30).

A very large observational study carried out in the United States on a female population reported an inverse relationship between the intakes of poultry and fish and the risk of developing coronary artery disease, as well as the absence of a clear relationship between red meat consumption and the same risk (31). The analysis of the data collected 26 years after the beginning of the study also reveals a positive correlation between the consumption levels of different protein sources (poultry, fish, and nuts) and health condition and survival (32). In particular, the substitution of one daily serving of red meat with a daily serving of poultry reduced CV risk by 19% (13% if red meat was substituted for reduced-fat dairy products and 24% if substituted for fish). The authors suggest that these benefits are a consequence of the reduction of heme iron and sodium and of the increase in polyunsaturated fats. The substitution of red meat for other protein sources such as poultry could therefore constitute an effective strategy to reduce coronary risk (30).

Possible underlying mechanisms linking the consumption levels of various meats and the risk of coronary disease can be extrapolated from the examination of the compositional differences between red and processed meat and white meat. In particular, saturated fats, cholesterol, and heme iron, which are higher in red versus white meats, have been described to be the key factors involved in atherosclerotic processes, CV risk factors, and chronic diseases such as hypertension, hypercholesterolemia, endothelial dysfunction, insulin resistance, and type 2 diabetes (4).

### Type 2 diabetes

A vast evidence demonstrates how lifestyle interventions can reduce the risk to develop type 2 diabetes by modifying several risk factors, including the excessive intake of fat, especially saturated fat (3). Studies dating back to the first half of the 20th century have highlighted that diabetes-related mortality increases in parallel to the increased Westernization of society, also characterized by a high consumption of meat (33, 34). More recent studies have confirmed the existence of a link between hyperinsulinemia and insulin resistance and the intake of saturated fat of animal origin (32, 35).

The literature on this topic has been systematically reviewed and has been the target of a meta-analysis based on 12 studies (36), characterized by a vast heterogeneity of results, mainly attributable to the variability in methodology applied across different cases. This meta-analysis has confirmed the relationship between the type 2 diabetes risk and the consumption of fatty and processed meats, against the absence of any link between total meat supply and the risk itself. Other studies have led to the same conclusion: the Health Professionals Follow-up Study (37), which followed 42,504 adults for 12 years and identified an elevated risk of diabetes with 5 weekly servings of processed meat (the risk increase was absent for poultry consumption); the EPIC-InterACT study (38), on more than 340,000 adults from eight European countries; and the most consistent meta-analysis, conducted by the Harvard group (39), on 20 studies for a total of 1,218,380 individuals (in this case, the association between risk and processed meat was maintained) and recently updated (40).

A recent re-elaboration of the data from the Interact study (41) has shown that the incidence of type 2 diabetes was higher in subjects with high total and animal protein intake levels, especially in females and in particular in those with a body mass index (BMI) over 30. Despite this finding, specific data concerning the consumption of poultry have confirmed the absence of a statistically significant relationship between an increasing weekly intake (100 g portions) of chicken and turkey and the development of the disease (32).

Benefits associated with poultry consumption have been described by the available literature on the effects of the intake of different types of foods on the progression of diabetes (10 prospective studies, for a total of 190,000 subjects). A dietary pattern comprising a high poultry intake, along with whole-grain cereals, fish, fruit, and vegetables, and a decrease in red meat consumption, processed foods, starches, and simple sugars seems to be effective in the management of the disease (42). The results from the EPIC study also sustain that following a healthier lifestyle and consuming poultry, as well as fruit, legumes, nuts, cereals, and vegetable oils, is correlated with a reduction of the mortality risk in a population of type 2 diabetic subjects, thus confirming that these patients can gain significant benefits from an overall change in lifestyle which includes white meat consumption (43). Of course, the available observations are not sufficient to support any independent association between the consumption of poultry alone and health. However, they clearly support the inclusion of poultry meat in healthy diets (44).

Amongst the nutritional factors that potentially increase the risk of diabetes, it was suggested that heme iron could play a role, because it increases oxidative stress and insulin resistance (3, 45). However, this hypothesis does not explain the negative effects of processed meats, in which heme iron is generally reduced (40).

Another confounding factor that should be taken into account is the difference between processed or cured meat and fresh meat, which have different concentrations of preservatives and sodium. It is estimated that, on average, processed meats contain approximately 400% more sodium and 50% more nitrates, weight by weight, than fresh meat (39). As concerns processed products, the temperature used in the preparation can also influence the impact on health: high temperatures, commonly used in the industrial meat manufacture, can induce the formation of heterocyclic amines and polycyclic aromatic hydrocarbons that could increase the risk of coronary artery disease, diabetes mellitus, and cancer (see below) (46). In particular, cross-sectional and prospective studies have highlighted the potential risks associated with the end products of glycation and lipoxidation present in processed foods, in addition to the mechanisms through which pancreatic cell function can be affected by the protein composition of cured meats (47).

### Cancer

Epidemiological studies conducted in populations with high or very high consumption levels of animal products show that excessive meat intake is a potential risk factor for specific cancer sites (48). The saturated fats, heme iron, sodium, and N-nitroso compounds contained in meat and the heterocyclic aromatic amines generated during cooking at high temperatures have been indicated as possible factors responsible for the positive meat–cancer relationship (49). The differences in the composition of poultry meat compared to red meat, in particular the lower amounts of potentially dangerous components, along with the content of others which, conversely, are nutritionally favorable (e.g. polyunsaturated fats), could at least partially explain the different impacts recorded for the risk of certain types of cancer across the two food categories. In general, increased consumption of red meat is associated with higher cancer risk, whereas white meat is considered moderately protective or neutral (50–52). Notably, red meat is characterized by a higher proportion of total fat (up to 20% vs. approximately 4% in lean poultry meat), especially of saturated fats, and a reduced content of polyunsaturated fats (11, 53).

According to the periodic report by the World Cancer Research Fund, individuals who usually consume animal products should privilege poultry and all types of fish over red meat. The consumption of the latter in the general population should not exceed 300 g of cooked red meat per week and, on an individual level, should be limited to a maximum of 500 g per week (equivalent to about 750 g of raw meat), limiting processed meats as much as possible (54).

Of all cancer types, those related to the digestive system are more commonly associated with the consumption of animal products. This observation emerged from studies conducted within populations with much higher consumption levels than those recommended by dietary guidelines (close to an excess); it was hypothesized that myoglobin supplied by red meat triggers precancerous lesions through the catalytic effect of heme iron on the production of carcinogenic N-nitroso compounds, and the development of cytotoxic and genotoxic aldehydes through lipid peroxidation (55). This hypothesis, which excludes the involvement of white meat in cancer risk, is confirmed across several meta-analyses.

For example, an analysis of the results of 13 studies with a total of over 500,000 subjects and 4,100 cases of oropharynx cancers (56) shows that these cancers’ risk increased for regular consumers of processed meat (3–6 servings per week on average), but not for consumers of other types of meat. This result confirms those obtained in a case-controlled study conducted by the Mario Negri Institute of Milan in the late 1990s across three provinces of Northern Italy (Milan, Padua, and Pordenone), showing that chicken and turkey meat were among those foods (along with pasta, raw vegetables, citrus fruits, and fruit in general) whose consumption correlates with a reduced risk of developing esophageal cancers (57). The effect was more pronounced (with an odd ratio equal to 0.4 and, hence, a relative risk reduction of about 60%) for the highest consumption levels. The relationship between reduced risk of esophageal cancer and white meat consumption, although not statistically significant, has been confirmed for both esophageal adenocarcinoma and esophageal squamous cell carcinoma in the meta-analysis of four cohort studies and 31 case–control studies carried on between 1990 and 2011 (51). The most recent systematic review of the literature confirms the inverse association between the number of poultry servings per week and the risk of esophageal carcinoma. This adds an important piece of information: considering only the highest quality European studies, high levels of poultry consumption have been shown to be associated with a total risk reduction of about 53% (52). The protective effect of chicken and turkey on esophageal tumors, similar to that on other cancers of the digestive system, has also been associated with nutritional status and lifestyle quality, which are generally higher in subjects who prefer these foods (58).

The negative influence of excess salt in preserved foods and processed meat on stomach cancer risk has been extensively described (59). In contrast, however, a few authors observed that the risk of such cancers is inversely associated with high levels of vegetable, fruit, vegetable oil, and poultry consumption, which would therefore exert a protective effect at the gastric mucosal level (60).

The survival of patients who are already affected by a colorectal cancer also seems to be negatively influenced by the intake of red and processed meat (61) with the prognosis in non-metastasized colorectal carcinoma being a notable example (62). As demonstrated by a meta-analysis (63), no relationship, on the other hand, was observed in cohort and epidemiological studies between chicken and turkey meat (assessed both separately and in the general context of white meat) and the risk of developing a colorectal cancer.

The available information concerning the effects of meat consumption on the risk of female breast cancer is rather heterogeneous. However, the absence of any significant relationship between poultry and this type of cancer has been assessed across various populations (64–67). This observation has been confirmed by a meta-analysis (1). In a prospective study conducted in a subpopulation of American nurses in premenopause at the time of recruitment, the incidence of invasive mammary carcinoma across 20 years of observation was inversely associated with poultry consumption (68). The assessment of the effects of different protein sources on the progression of the disease enabled one to estimate that the substitution of one daily portion of red meat with one of poultry could reduce the risk of breast cancer by approximately 17% in general and by 24% in postmenopausal women.

Both cohort and case–control studies tend to exclude the presence of a relationship between meat consumption and ovarian carcinoma, hypothesized on the basis of the results of ecological studies, most probably influenced by confounding factors (69). Despite a limited number of high-quality studies, both a meta-analysis of prospective studies (70) and the EPIC study conducted on more than 350,000 European women (71) have concluded that no type of meat, including white meat, has a significant effect on the incidence of ovarian tumors. In addition, analysis of the impact of various nutrients led to the exclusion of any association between this type of cancer and total fats, saturated fats, and more specifically fat from meat (72); only a positive correlation with industrially produced unsaturated trans fats was observed.

Literature evidence remains inconsistent for endometrial cancer (73); total, lymphocytic, and myeloid leukemia (74); and hepatocellular carcinoma (75). However, a high intake of poultry could reduce the risk of lung cancer by approximately 10% according to a meta-analysis on 23 case–control and 11 cohort studies (6).

## Poultry meat consumption and the overall quality of the diet in various stages of life

The relevance of poultry meat for humans has been evaluated by FAO which, in a recent document, states that ‘the human population benefits greatly from poultry meat and eggs, which provide food containing high-quality protein, and a low level of fat with a desirable fatty acid profile’. In particular, these foods, which are widely available and relatively inexpensive, might be particularly useful in developing countries, where they can help to meet shortfalls in essential nutrients for impoverished people. The incidence of several common metabolic diseases associated with deficiencies in critical dietary minerals, vitamins, and amino acids can be reduced by the contribution of poultry products, which are rich in all essential nutrients except vitamin C (76). Moreover, poultry meat consumption also contributes to the overall quality of the diet in specific ages and conditions. For example, in the period prior to conception, during pregnancy, and up to the end of breastfeeding, the quality of the maternal diet is amongst the factors affecting the health of both the mother and the infant.

The guidelines relative to this delicate period generally refer to the variety of foods to be consumed, the number of meals (4, 5), consumption modalities (chewing slowly), and adequate hydration. Well-cooked lean meats (e.g. chicken and turkey) are to be privileged during pregnancy (77).

The required dietary intakes of vitamins (A, D, C, B6, B12, and folic acid), minerals (calcium, iron, and phosphorus), and essential fatty acids all increase during gestation (78). Poultry, which is a good source of some of these nutrients, and also of the essential linoleic and alpha-linolenic fatty acids, can represent a good nutrient source.

At the same time, poultry meat consumption can also help in reducing salt intake and, therefore, that of sodium, which should be as moderate as possible for both the mother and her child.

Chicken and turkey also are valuable components of a balanced diet during growth, when their meat can fulfill specific growth requirements, due to its high protein content (characterized by the presence of the essential amino acids lysine, histidine, and arginine) and moderate fats (especially after skin removal), which are prevalently unsaturated as opposed to saturated and are highly bioavailable; vitamins (e.g. B group vitamins); and minerals (e.g. iron) (79).

In particular, among meats recommended for weaning, chicken and turkey (together with fish and lamb) are the easiest to puree. Moreover, baby foods containing these meats are easily digestible and are characterized by a low allergenicity (80).

The level of minerals, in particular iron, in poultry meats makes them suitable for even the most advanced stages of growth, such as adolescence, during which greater autonomy can increase the risk of an unbalanced nutrient intake (81).

The geriatric age is another life period in which wellbeing is more strictly associated with diet and lifestyle, as demonstrated by epidemiological observations in several countries (82). The increased availability of good-quality foods has eradicated most nutritional deficiencies in the elderly and has contributed to the increase in healthy life expectancy. Nonetheless, nutritional balance cannot be taken for granted in this stage of life, even in developed Western countries.

An increased required intake of specific nutrients such as calcium (useful in the control of bone mass loss, particularly in the female population) and protein goes in parallel with a reduction in total caloric needs (essentially due to the age-associated decrease in physical activity). Many studies have found that an adequate intake of protein in old age helps fight against physiological age-related sarcopenia, the gradual decrease in muscle mass with serious consequences in terms of movement and individual autonomy (83). Specifically, poultry meat, which is a good-quality protein source that is characterized by high digestibility and chewability, especially when prepared using light cooking methods, is particularly important for the elderly, who often have to deal with digestive disorders or chewing difficulties.

Finally, it must be noted that poultry meat is significantly less expensive compared to other meats: a non-negligible aspect in the context of elderly nutrition, as this population group is subject to generally lower incomes and is at risk of following an unbalanced diet due to financial limitations.

## Conclusions

- Poultry meats are characterized by a good overall nutritional profile. Their high-biological-value protein, vitamin, and mineral content associated with a low fat content (most of which is composed of unsaturated fatty acids) enables these meats to be optimally incorporated into the diet at all ages.
- Cross-sectional and prospective epidemiological studies support this view, showing that adequate consumption of chicken meat can facilitate the control of body weight (especially due to its high protein content), with a neutral or positive effect on the risk of developing the main degenerative diseases typical of our society (i.e. CV disease, diabetes, and cancer).
- Chicken meat, because of its favorable nutritional profile, can play an important role for individuals in specific age groups (pregnant women, children, and the elderly).
- Consumption of these meats in the context of a balanced diet and alongside an adequate intake of protein-based foods, including plant-based ones, would likely contribute to the overall quality of the diet in the population.

## References

[1] Missmer SA, Smith-Warner SA, Spiegelman D, Yaun SS, Adami HO, Beeson WL (2002). Meat and dairy food consumption and breast cancer: a pooled analysis of cohort studies. Int J Epidemiol.

[2] Chen GC, Lv DB, Pang Z, Liu QF (2013). Red and processed meat consumption and risk of stroke: a meta-analysis of prospective cohort studies. Eur J Clin Nutr.

[3] Ley SH, Sun Q, Willett WC, Eliassen AH, Wu K, Pan A (2014). Associations between red meat intake and biomarkers of inflammation and glucose metabolism in women. Am J Clin Nutr.

[4] Abete I, Romaguera D, Vieira AR, Lopez de Munain A, Norat T (2014). Association between total, processed, red and white meat consumption and all-cause, CVD and IHD mortality: a meta-analysis of cohort studies. Br J Nutr.

[5] Sinha R, Cross AJ, Graubard BI, Leitzmann MF, Schatzkin A (2009). Meat intake and mortality: a prospective study of over half a million people. Arch Intern Med.

[6] Yang WS, Wong MY, Vogtmann E, Tang RQ, Xie L, Yang YS (2012). Meat consumption and risk of lung cancer: evidence from observational studies. Ann Oncol.

[7] Oostindjer M, Alexander J, Amdam GV, Andersen G, Bryan NS, Chen D (2014). The role of red and processed meat in colorectal cancer development: a perspective. Meat Sci.

[8] World Health Organization European Region (2003). Food based dietary guidelines in the WHO European Region.

[9] Bernstein AM, Sun Q, Hu FB, Stampfer MJ, Manson JE, Willett WC (2010). Major dietary protein sources and risk of coronary heart disease in women. Circulation.

[10] Millen BE, Wolongevicz DM, de Jesus JM, Nonas CA, Lichtenstein AH (2014). 2013 American Heart Association/American College of Cardiology Guideline on Lifestyle Management to Reduce Cardiovascular Risk: practice opportunities for registered dietitian nutritionists. J Acad Nutr Diet.

[11] Gnagnarella P, Salvini S, Parpinel M Food composition database for epidemiological studies in Italy. European Institute of Oncology 2008.

[12] Lofgren PA, Caballero B, Allen L, Prentice A (2005). Meat, poultry and meat products. Encyclopedia of human nutrition.

[13] FAO/WHO/ÚNU Expert Consultation (1985). Endogenous recoveries of true ileal digestibilities of amino acids in newly weaned piglets fed diets with protease-treated soybean meal. Energy and protein requirements. Technical Report Series 724.

[14] EFSA Panel on Dietetic Products, Nutrition and Allergies (NDA) (2012). Scientific opinion on dietary reference values for protein. EFSA J.

[15] Levine ME, Suarez JA, Brandhorst S, Balasubramanian P, Cheng CW, Madia F (2014). Low protein intake is associated with a major reduction in IGF-1, cancer, and overall mortality in the 65 and younger but not older population. Cell Metab.

[16] Hibbeln JR, Nieminen LR, Blasbalg TL, Riggs JA, Lands WE (2006). Healthy intakes of n-3 and n-6 fatty acids: estimations considering worldwide diversity. Am J Clin Nutr.

[17] Ian Givens D, Gibbs RA (2008). Current intakes of EPA and DHA in European populations and the potential of animal-derived foods to increase them. Proc Nutr Soc.

[18] Lombardi-Boccia G, Martinez-Dominguez B, Aguzzi A (2006). Total heme and non-heme iron in raw and cooked meats. J Food Sci.

[19] Daniel CR, Cross AJ, Koebnick C, Sinha R (2011). Trends in meat consumption in the USA. Public Health Nutr.

[20] Linseisen J, Kesse E, Slimani N, Bueno-De-Mesquita HB, Ocké MC, Skeie G (2002). Meat consumption in the European Prospective Investigation into Cancer and Nutrition (EPIC) cohorts: results from 24-hour dietary recalls. Public Health Nutr.

[21] Leclercq C, Arcella D, Piccinelli R, Sette S, Le Donne C, Turrini A (2009). The Italian National Food Consumption Survey INRAN-SCAI 2005–06: main results in terms of food consumption. Public Health Nutr.

[22] Clifton PM (2011). Protein and coronary heart disease: the role of different protein sources. Curr Atheroscler Rep.

[23] Halton TL, Hu FB (2004). The effects of high protein diets on thermogenesis, satiety and weight loss: a critical review. J Am Coll Nutr.

[24] Te Morenga L, Mann J (2012). The role of high-protein diets in body weight management and health. Br J Nutr.

[25] Paoli A (2014). Ketogenic diet for obesity: friend or foe?. Int J Environ Res Public Health.

[26] Astrup A, Raben A, Geiker N (2014). The role of higher protein diets in weight control and obesity-related comorbidities. Int J Obes (Lond).

[27] Westerterp-Plantenga MS, Nieuwenhuizen A, Tomé D, Soenen S, Westerterp KR (2009). Dietary protein, weight loss, and weight maintenance. Annu Rev Nutr.

[28] Promintzer M, Krebs M (2006). Effects of dietary protein on glucose homeostasis. Curr Opin Clin Nutr Metab Care.

[29] Vergnaud AC, Norat T, Romaguera D, Mouw T, May AM, Travier N (2010). Meat consumption and prospective weight change in participants of the EPIC-PANACEA study. Am J Clin Nutr.

[30] Hu FB (2005). Protein, body weight, and cardiovascular health. Am J Clin Nutr.

[31] Hu FB, Stampfer MJ, Manson JE, Ascherio A, Colditz GA, Speizer FE (1999). Dietary saturated fats and their food sources in relation to the risk of coronary heart disease in women. Am J Clin Nutr.

[32] Feskens EJ, Sluik D, van Woudenbergh GJ (2013). Meat consumption, diabetes, and its complications. Curr Diab Rep.

[33] Zimmet P, Alberti KG, Shaw J (2001). Global and societal implications of the diabetes epidemic. Nature.

[34] Lee JE, McLerran DF, Rolland B, Chen Y, Grant EJ, Vedanthan R (2013). Meat intake and cause-specific mortality: a pooled analysis of Asian prospective cohort studies. Am J Clin Nutr.

[35] Pan A, Sun Q, Bernstein AM, Schulze MB, Manson JE, Willett WC (2011). Red meat consumption and risk of type 2 diabetes: 3 cohorts of US adults and an updated meta-analysis. Am J Clin Nutr.

[36] Aune D, Ursin G, Veierød MB (2009). Meat consumption and the risk of type 2 diabetes: a systematic review and meta-analysis of cohort studies. Diabetologia.

[37] van Dam RM, Willett WC, Rimm EB, Stampfer MJ, Hu FB (2002). Dietary fat and meat intake in relation to risk of type 2 diabetes in men. Diabetes Care.

[38] InterAct Consortium (2014). Adherence to predefined dietary patterns and incident type 2 diabetes in European populations: EPIC-InterAct Study. Diabetologia.

[39] Micha R, Wallace SK, Mozaffarian D (2010). Red and processed meat consumption and risk of incident coronary heart disease, stroke, and diabetes mellitus: a systematic review and meta-analysis. Circulation.

[40] Micha R, Michas G, Mozaffarian D (2012). Unprocessed red and processed meats and risk of coronary artery disease and type 2 diabetes – an updated review of the evidence. Curr Atheroscler Rep.

[41] van Nielen M, Feskens EJ, Mensink M, Sluijs I, Molina E, Amiano P (2014). InterAct consortium. Dietary protein intake and incidence of type 2 diabetes in Europe: the EPIC-InterAct case-cohort study. Diabetes Care.

[42] Esposito K, Kastorini CM, Panagiotakos DB, Giugliano D (2010). Prevention of type 2 diabetes by dietary patterns: a systematic review of prospective studies and meta-analysis. Metab Syndr Relat Disord.

[43] Sluik D, Boeing H, Li K, Kaaks R, Johnsen NF, Tjønneland A (2014). Lifestyle factors and mortality risk in individuals with diabetes mellitus: are the associations different from those in individuals without diabetes?. Diabetologia.

[44] Esposito K, Chiodini P, Maiorino MI, Bellastella G, Panagiotakos D, Giugliano D (2014). Which diet for prevention of type 2 diabetes? A meta-analysis of prospective studies. Endocrine.

[45] Kim Y, Keogh J, Clifton P (2015). A review of potential metabolic etiologies of the observed association between red meat consumption and development of type 2 diabetes mellitus. Metabolism.

[46] Birlouez-Aragon I, Saavedra G, Tessier FJ, Galinier A, Ait-Ameur L, Lacoste F (2010). A diet based on high-heat-treated foods promotes risk factors for diabetes mellitus and cardiovascular diseases. Am J Clin Nutr.

[47] White DL, Collinson A (2013). Red meat, dietary heme iron, and risk of type 2 diabetes: the involvement of advanced lipoxidation endproducts. Adv Nutr.

[48] Kushi LH, Doyle C, McCullough M, Rock CL, Demark-Wahnefried W, Bandera EV (2012). American Cancer Society 2010 Nutrition and Physical Activity Guidelines Advisory Committee. American Cancer Society Guidelines on nutrition and physical activity for cancer prevention: reducing the risk of cancer with healthy food choices and physical activity. CA Cancer J Clin.

[49] Turesky RJ (2007). Formation and biochemistry of carcinogenic heterocyclic aromatic amines in cooked meats. Toxicol Lett.

[50] Bingham SA (1999). High-meat diets and cancer risk. Proc Nutr Soc.

[51] Salehi M, Moradi-Lakeh M, Salehi MH, Nojomi M, Kolahdooz F (2013). Meat, fish, and esophageal cancer risk: a systematic review and dose-response meta-analysis. Nutr Rev.

[52] Zhu HC, Yang X, Xu LP, Zhao LJ, Tao GZ, Zhang C (2014). Meat consumption is associated with esophageal cancer risk in a meat- and cancer-histological-type dependent manner. Dig Dis Sci.

[53] Genkinger JM, Hunter DJ, Spiegelman D, Anderson KE, Beeson WL, Buring JE (2006). A pooled analysis of 12 cohort studies of dietary fat, cholesterol and egg intake and ovarian cancer. Cancer Causes Control.

[54] World Cancer Research Fund/American Institute for Cancer Research (2007). Food, nutrition, physical activity, and the prevention of cancer: a global perspective.

[55] Bastide NM, Pierre FH, Corpet DE (2011). Heme iron from meat and risk of colorectal cancer: a meta-analysis and a review of the mechanisms involved. Cancer Prev Res (Phila).

[56] Xu J, Yang XX, Wu YG, Li XY, Bai B (2014). Meat consumption and risk of oral cavity and oropharynx cancer: a meta-analysis of observational studies. PLoS One.

[57] Bosetti C, La Vecchia C, Talamini R, Simonato L, Zambon P, Negri E (2000). Food groups and risk of squamous cell esophageal cancer in northern Italy. Int J Cancer.

[58] Flood A, Rastogi T, Wirfält E, Mitrou PN, Reedy J, Subar AF (2008). Dietary patterns as identified by factor analysis and colorectal cancer among middle-aged Americans. Am J Clin Nutr.

[59] Dias-Neto M, Pintalhao M, Ferreira M, Lunet N (2010). Salt intake and risk of gastric intestinal metaplasia: systematic review and meta-analysis. Nutr Cancer.

[60] Ji BT, Chow WH, Yang G, McLaughlin JK, Zheng W, Shu XO (1998). Dietary habits and stomach cancer in Shanghai, China. Int J Cancer.

[61] van Meer S, Leufkens AM, Bueno-de-Mesquita HB, van Duijnhoven FJ, van Oijen MG, Siersema PD (2013). Role of dietary factors in survival and mortality in colorectal cancer: a systematic review. Nutr Rev.

[62] McCullough ML, Gapstur SM, Shah R, Jacobs EJ, Campbell PT (2013). Association between red and processed meat intake and mortality among colorectal cancer survivors. J Clin Oncol.

[63] Xu B, Sun J, Sun Y, Huang L, Tang Y, Yuan Y (2013). No evidence of decreased risk of colorectal adenomas with white meat, poultry, and fish intake: a meta-analysis of observational studies. Ann Epidemiol.

[64] Männistö S, Dixon LB, Balder HF, Virtanen MJ, Krogh V, Khani BR (2005). Dietary patterns and breast cancer risk: results from three cohort studies in the DIETSCAN project. Canc Causes Contr.

[65] Hu J, La Vecchia C, DesMeules M, Negri E, Mery L (2008). Canadian cancer registries epidemiology research group meat and fish consumption and cancer in Canada. Nutr Cancer.

[66] Zhang CX, Ho SC, Chen YM, Lin FY, Fu JH, Cheng SZ (2009). Meat and egg consumption and risk of breast cancer among Chinese women. Canc Causes Contr.

[67] Pala V, Krogh V, Berrino F, Sieri S, Grioni S, Tjønneland A (2009). Meat, eggs, dairy products, and risk of breast cancer in the European Prospective Investigation into Cancer and Nutrition (EPIC) cohort. Am J Clin Nutr.

[68] Farvid MS, Cho E, Chen WY, Eliassen AH, Willett WC (2014). Dietary protein sources in early adulthood and breast cancer incidence: prospective cohort study. BMJ.

[69] Kolahdooz F, van der Pols JC, Bain CJ, Marks GC, Hughes MC, Whiteman DC (2010). Australian Cancer Study (Ovarian Cancer) and the Australian Ovarian Cancer Study Group. Meat, fish, and ovarian cancer risk: results from 2 Australian case-control studies, a systematic review, and meta-analysis. Am J Clin Nutr.

[70] Wallin A, Orsini N, Wolk A (2011). Red and processed meat consumption and risk of ovarian cancer: a dose-response meta-analysis of prospective studies. Br J Cancer.

[71] Schulz M, Nöthlings U, Allen N, Onland-Moret NC, Agnoli C, Engeset D (2007). No association of consumption of animal foods with risk of ovarian cancer. Cancer Epidemiol Biomarkers Prev.

[72] Gilsing AM, Weijenberg MP, Goldbohm RA, van den Brandt PA, Schouten LJ (2011). Consumption of dietary fat and meat and risk of ovarian cancer in the Netherlands Cohort Study. Am J Clin Nutr.

[73] Bandera EV, Kushi LH, Moore DF, Gifkins DM, McCullough ML (2007). Consumption of animal foods and endometrial cancer risk: a systematic literature review and meta-analysis. Canc Causes Contr.

[74] Saberi Hosnijeh F, Peeters P, Romieu I, Kelly R, Riboli E, Olsen A (2014). Dietary intakes and risk of lymphoid and myeloid leukemia in the European Prospective Investigation into Cancer and Nutrition (EPIC). Nutr Cancer.

[75] Fedirko V, Trichopolou A, Bamia C, Duarte-Salles T, Trepo E, Aleksandrova K (2013). Consumption of fish and meats and risk of hepatocellular carcinoma: the European Prospective Investigation into Cancer and Nutrition (EPIC). Ann Oncol.

[76] Ravindran V (2013). FAO Poultry Development Review. Poultry Feed Availability and Nutrition in Developing Countries: Main Ingredients Used in Poultry Feed Formulations. http://www.fao.org/docrep/019/i3531e/i3531e.pdf.

[77] WHO (2002). Complementary feeding. Report of the global consultation convened jointly by the Department of Child and Adolescent Health and Development and the Department of Nutrition for Health and Development and Summary of guiding principles for complementary feeding of the breastfed child.

[78] Allen LH (2005). Multiple micronutrients in pregnancy and lactation: an overview. Am J Clin Nutr.

[79] Gidding SS, Dennison BA, Birch LL, Daniels SR, Gillman MW, Lichtenstein AH (2005). Dietary recommendations for children and adolescents: a guide for practitioners: consensus statement from the American Heart Association. Circulation.

[80] Fiocchi A, Assa'ad A, Bahna S (2006). Adverse reactions to foods committee, American College of Allergy, Asthma and Immunology. Food allergy and the introduction of solid foods to infants: a consensus document. Adverse reactions to foods committee, American College of Allergy, Asthma and Immunology. Ann Allergy Asthma Immunol.

[81] Elmadfa I, Weichselbaum E (2005). European nutrition and health report 2004. Vol. 58.

[82] Volkert D (2013). Malnutrition in older adults – urgent need for action: a plea for improving the nutritional situation of older adults. Gerontology.

[83] Phillips SM (2012). Nutrient-rich meat proteins in offsetting age-related muscle loss. Meat Sci.

